# Immunotherapeutic Approaches in Triple-Negative Breast Cancer: State of the Art and Future Perspectives

**DOI:** 10.1155/2020/8209173

**Published:** 2020-11-04

**Authors:** Karima Oualla, Loay Kassem, Lamiae Nouiakh, Lamiae Amaadour, Zineb Benbrahim, Samia Arifi, Nawfel Mellas

**Affiliations:** ^1^Medical Oncology Department, Hassan II University Hospital, Sidi Mohamed Ben Abdellah University, Fes, Morocco; ^2^Clinical Oncology Department, Kasr Al-Ainy School of Medicine, Cairo University, Giza, Egypt

## Abstract

Triple-negative breast cancer (TNBC) is characterized by the absence of estrogen receptor (ER), progesterone receptor (PR), and human epidermal growth factor receptor 2 (HER2). It accounts for 15%–20% of all breast cancers and is associated with an aggressive evolution and poor outcomes with the majority of recurrences and deaths occurring in the first 5 years. Chemotherapy remains the mainstay of treatment in the absence of effective targets, but the good understanding of immune tumor microenvironment, the identification of immune-related targets, and the role of tumor-infiltrating lymphocytes (TILs) in TNBC has allowed to develop promising immunotherapeutic strategies for this unique subset of breast cancer. Recently, immunotherapy is being extensively explored in TNBC and clinical trials have shown promising results. In this article, we tried to explain the rationale and mechanisms of targeting the immune system in TNBC, to report the results from recent clinical trials that put immunotherapy as a new standard of care in TNBC in addition to ongoing trials and future directions in the next decade.

## 1. Overview

Triple-negative breast cancer (TNBC) is a molecular subtype of breast cancer characterized by the absence of expression of estrogen receptor (ER), progesterone receptor (PR), and human epidermal growth factor receptor 2 (HER2) [1]. TNBC tends to occur more in young women (<40 years old) and typically presents with aggressive biology including high-grade invasive ductal carcinomas and high proliferative rate [2]. It also presents a particular pattern of metastases with higher rates of visceral and brain metastases, and poor outcomes with early recurrences and shorter survival [2].

At the molecular level, distinct intrinsic subtypes of TNBC were distinguished using gene expression signatures [3]. Gene expression profiling of TNBCs has identified six molecular subtypes including two basal-like subtypes (BL1 and BL2), mesenchymal (M), mesenchymal stem-like (MSL), immunomodulatory (IM), and a luminal androgen receptor (LAR) [4, 5]. This genomic profiling allowed conducting several researches aiming at developing more personalized treatments for TNBC patients.

Therapeutically, for many decades, cytotoxic chemotherapy was the mainstay treatment in the absence of actionable targets with short survival [6]. Then recently, it has been shown that the immune system has an important role in tumor initiation and progression of breast cancer and also in the destruction of cancer cells [7]. The genetic and epigenetic alterations in TNBC lead to tumor-associated antigens that allow the immune system to recognize tumor cells from normal cells. The immune system blocks the development and progression of cancer cells via tumor-directed immune responses involving mainly T lymphocytes.

Active immunotherapy using immune checkpoint inhibitors (ICIs) acts by enhancing the activity of the immune system via disrupting negative immune regulators to enhance the immune response [8]. Therefore, different ICIs have been first explored in cancers considered as highly immunogenic such as melanoma, lung cancer, and renal cancer. ICIs have shown impressive results which made them new standards of care in the treatment of early and advanced stages of different cancers including bronchial cancers, melanomas, and urothelial cancers. These impressive results led to investigating the role of ICIs in breast cancer particularly in the TNBC subtype.

## 2. Immunogenicity of TNBC

Unlike melanoma, lung cancer, and kidney cancer, breast cancer was considered as a nonimmunogenic cancer for long decades with low T cell infiltration. However, in contrast to the other breast cancer subtypes, TNBC is characterized by higher tumor immune infiltrate and higher degree of stromal and tumor-infiltrating lymphocytes (TILs) [9]. Several studies have shown that the TILs were associated with better prognosis and higher response to therapy in breast cancer especially in TNBC [10, 11].

Additionally, further studies have suggested activation of inhibitory immune checkpoints (as CTLA-4 and PD-1/PD-L1 axis) in TNBCs with higher PD-L1 expression in comparison with luminal subtypes. The inhibitory action of PD-1 bound to its ligand (PD-L1) suppresses the immune response in cancer cells. Therefore, the upregulation of PD-L1 expression in tumor cells allows an evasion of the tumor cells from the immune system detection, which subsequently leads to tumor progression [12].

PD-L1 expression inhibits different immune cells in the tumor microenvironment including T cells, B cells, natural killer cells, dendritic cells, and macrophages, suggesting that PD-1 expression is a mechanism of restriction of immunity, provided through the innate and adaptive immune system. PD-1 has a major role in the negative regulation of T cell activity by blocking T cells and modulating immune response [13].

Moreover, TNBC is characterized by a high mutational burden that provides genomic instability and subsequently leads to higher production of neoantigens which make TNBC more immunogenic than other breast cancer subtypes.

Several factors intervene in the antitumor immune response. The cytotoxic CD8-positive T lymphocytes, type 1 macrophages, and intratumoral B cells play a crucial role in the antitumor microenvironment. Additionally, FOXP3^+^CD4^+^ regulatory T cells (Tregs) are mediators of immune tolerance, and therefore, they are associated with poor outcomes [13]. The PD-L1 was reported to be expressed in around 20% to 30% of TNBC and was correlated with aggressive characteristics including higher grade and high proliferation rate. PD-1 interacts with its ligand PD-L1, and this interaction on T cells is a major mechanism of tumor immune evasion and leads to the suppression of antitumor immunity by exerting a negative regulation on T cells, cytolytic activity, and production of cytokine. Subsequently, the blockade of these targets leads to increasing the antitumor immune response by the blockade of immune-regulating proteins that downregulate the immune system [14].

CTLA4 also has an important role in regulating immune responses early in the process of T cell activation. Therefore, its inhibition by monoclonal antibodies blocks the interaction between T cells and the receptor via CD28 on its cell surface. This blockade increases the ratio of CD8^+^ T cells to Foxp3^+^ T regulatory cells, promotes the antitumor activity of CD8^+^ T cells, and blocks the suppressive function of T regulatory cells [13, 14].

These results, put together, show that immunotherapy is a promising modality in TNBC, and the use of anti-PD-1/PD-L1 in the treatment of TNBC must receive much attention.

## 3. Clinical Trials with Immune Checkpoint Inhibitors in TNBC (Table 1)

### 3.1. PD-1 Inhibitors

#### 3.1.1. Pembrolizumab

Pembrolizumab, a PD-1 inhibitor, has been evaluated as monotherapy in PD-L1-positive heavily pretreated metastatic TNBC patients [15]. In this phase 1b trial, 47% of patients had received more than 3 lines of treatment and 21.9% had received 5 or more treatments. The results revealed an objective response rate (ORR) of 18.5%, a partial response in 14.8% of patients, a complete response (CR) in 3.7% of cases, and 25.9% had a stable disease (SD). The overall survival (OS) rate at 2 years was 22% [15]. It can be explained by the maintained disease control seen in good responders to therapy.

Another phase single-arm multicohort II study (KEYNOTE-086) has investigated the role of pembrolizumab in monotherapy in pretreated metastatic TNBC [16]. Cohort A included 170 TNBC patients who received one or more systemic therapy. The endpoint was to assess the efficacy and the safety of pembrolizumab independently of PD-L1 expression. The primary endpoints were the ORR in the total and PD-L1-positive populations and safety. Sixty percent of patients had PD-L1-positive tumors. After a median follow-up of 11.9 months, the ORR was 5% and the disease control rate was 8%. The response rate was not impacted by PD-L1. The median PFS was 2.0 months with an estimated 6-month PFS rate of 12% [16]. Cohort B of the same study included 52 patients and investigated the safety and efficacy of pembrolizumab in patients with TNBC with no prior systemic treatment for tumors with PD-L1-positive tumors defined by an IHC-based composite score. The ORR was 23% after a median follow-up of 7 months, and the median PFS was 2.1 months (95% CI 2.0-3.9); the estimated 6-month PFS rate was 29%, and the median duration of response was 8.4 months [17]. The improved response in cohort B may be a result of the use of pembrolizumab as a first-line treatment and the selection of only PD-L1^+^ tumors as an inclusion criterion.

A phase Ib/II trial has assessed the combination of pembrolizumab with eribulin mesylate in patients with metastatic disease, treated with at least 2 prior lines of chemotherapy [18].

The findings revealed an ORR of 25.6% in the 82 evaluated patients and 30.5% of the clinical benefit rate. Response was regardless of PD-L1 expression (25.7% and 25.0% in the PD-L1-positive and PD-L1-negative cohorts, respectively) [18]. The combination also resulted in improved PFS and OS independently of PD-L1 status, and the safety profile was comparable to monotherapy. Further exploration of this combination is needed.

KEYNOTE-119 is a phase III study that has evaluated pembrolizumab versus single-agent chemotherapy per investigator's choice (capecitabine, eribulin, gemcitabine, or vinorelbine) in 662 patients with metastatic TNBC who progressed on 1 or more lines of chemotherapy. The study did not meet its prespecified primary endpoint of superior OS compared to standard chemotherapy. The trial did not show any new safety concerns [19].

Another global phase III trial (KEYNOTE-355, NCT02819518) has assessed the combination of pembrolizumab with chemotherapy versus placebo with chemotherapy in patients with previously untreated, locally recurrent, inoperable TNBC. Pembrolizumab plus chemotherapy significantly improved PFS vs. chemotherapy alone when CPS ≥ 10 which is one of the primary objectives. Despite the boundary for a statistically significant benefit of the combination in patients with CPS ≥ 1 tumors was not met and formal testing in ITT was not performed, the pembrolizumab treatment efficacy was higher with PD-L1 enrichment. OS follow-up is ongoing, and no new safety concerns were reported [20].

The promising results in metastatic stages of TNBC provided enough evidence to conduct studies with pembrolizumab in early stages. Among them, the I-SPY 2 phase II multicenter trial evaluated the addition of pembrolizumab to standard neoadjuvant chemotherapy based on paclitaxel followed by doxorubicin and cyclophosphamide. This study showed that the combination [19] was likely to result in a significant improvement of pCR in TNBC (60% vs. 20%) [21].

More recently, the KEYNOTE-522 phase III study has assessed the addition of pembrolizumab [19] to neoadjuvant chemotherapy for stages IIa to IIIb TNBC [22]. Patients were randomized 2 : 1 to pembrolizumab or placebo. Both arms were given with 4 cycles of paclitaxel+carboplatin and then with 4 cycles of doxorubicin or epirubicin+cyclophosphamide. In adjuvant setting after curative surgery, patients received pembrolizumab or placebo for 9 cycles or until relapse or unacceptable toxicity. Primary endpoints were the pathologic complete remission rate (pCR) and event-free survival (EFS).

After a median follow-up of 15.5 months, adding pembrolizumab significantly improved pCR compared to chemotherapy alone: 64.8% vs. 51.2% (*p* = 0.00055). For pembrolizumab vs. placebo, pCR was 68.9% vs. 54.9% in the PD-L1^+^ population and 45.3% vs. 30.3% in the PD-L1 population. Additionally, the pembrolizumab arm also showed a favorable trend in EFS (HR = 0.63 [95% CI 0.43-0.93]). Regarding safety, grade 3 or higher treatment-related adverse event rates were 78.0% in the pembrolizumab plus chemotherapy group and 73% in the placebo+chemotherapy group (death incidence, 0.4% vs. 0.3%, respectively) [22].

Another ongoing phase III trial (SWOG-S1418, BR006; NCT02954874) is evaluating the efficacy and safety of pembrolizumab as adjuvant therapy for TNBC with ≥1 cm residual invasive cancer or positive lymph nodes (ypN^+^) after neoadjuvant chemotherapy.

#### 3.1.2. Nivolumab

Nivolumab was assessed in several phase I and II trials of TNBC patients. The TONIC trial is an adaptive phase II randomized noncomparative trial that evaluated nivolumab in patients with metastatic TNBC after induction treatment including radiation, low-dose doxorubicin, metronomic cyclophosphamide, and cisplatin [23]. The objective response rate (ORR) per RECIST v1.1 with nivolumab for the whole cohort was 22% and 24% for iRECIST, which included 1 (2%) CR, and 11 (22%) PR. Additionally, stable disease (SD) lasting more than 24 weeks was achieved in 1 (2%) patient, which resulted in a 26% clinical benefit rate (CBR = CR + PR + SD > 24 weeks). The median duration of response was 9 months (95% confidence interval (CI) 5.5-NA). Preliminary analyses showed that the response rate might be higher after induction therapy with doxorubicin or cisplatin and those patients with higher leukocyte infiltration and CD8 T cell counts were better responders to treatment [23].

Another ongoing phase II study is evaluating carboplatin with or without nivolumab in metastatic TNBC (NCT03414684).

### 3.2. PD-L1 Inhibitors

#### 3.2.1. Atezolizumab

Atezolizumab is a monoclonal antibody that binds selectively to PD-L1 on immune cells or tumor cells to prevent interactions with the PD-1 receptor or B7-1. A phase I study of single-agent atezolizumab included 116 patients with metastatic TNBC irrespective of prior therapy or PD-L1 status [24]. The primary endpoints were safety and tolerability. The ORR was 10% in the overall population and 24% in those receiving atezolizumab as first-line treatment. Interestingly, the median duration of response was 21 months (range, 3 to ≥38). Of note, liver metastases, LDH levels, tumor burden, and performance status were associated with worse outcomes. Treatment-related adverse events were mostly grades 1–2 (in 79% of cases) with the most common being fever, fatigue and nausea, diarrhea, and pruritus [24].

Important results were presented from the phase III IMpassion130 trial. This study included 902 patients with treatment-naïve metastatic TNBC who were randomly assigned to receive nab-paclitaxel alone or in combination with atezolizumab. The combination reduced the risk of disease progression or death by 20% in all patients and by 38% in the subgroup expressing PD-L1 which accounted for 41% of all patients [25].

After a median follow-up of slightly more than 1 year, the median progression-free survival (PFS) was 7.2 months with the combination compared with 5.5 months for placebo plus nab-paclitaxel in the intention-to-treat analysis (hazard ratio (HR) for progression or death = 0.80; *p* = 0.002). Patients with PD-L1-positive tumors who received the combination had a 38% reduction in the risk of progression and death compared with nab-paclitaxel alone (HR = 0.62; 7.5 vs. 5.0 months; *p* < 0.001).

At first interim analysis, in the intent-to-treat group, median OS was 21.3 months with the combination compared with 17.6 months for placebo plus nab-paclitaxel (HR = 0.84; *p* = 0.08). Among those with PD-L1-positive tumors, the median overall survival was 25.0 months compared with 15.5 months, respectively. The safety of atezolizumab plus nab-paclitaxel was consistent with the known toxic effects of each agent [25].

After these results, the Food and Drug Administration (FDA) granted accelerated approval to atezolizumab in March 2019, and atezolizumab in combination with nab-paclitaxel became the new upfront standard of care in the subset of patients with unresectable locally advanced or metastatic PD-L1-positive TNBC.

Another phase III trial is evaluating atezolizumab in combination with paclitaxel compared with placebo with paclitaxel for patients with previously untreated inoperable locally advanced or metastatic TNBC (NCT03125902) [26]. In the neoadjuvant setting, a phase III randomized study is investigating the efficacy and safety of atezolizumab in combination with neoadjuvant anthracycline/nab-paclitaxel-based chemotherapy compared with placebo and chemotherapy (NCT03197935) [27]. Another phase III neoadjuvant trial is studying the efficacy of atezolizumab in locally advanced TNBC patients undergoing treatment with nab-paclitaxel and carboplatin. The addition of atezolizumab to neoadjuvant chemotherapy did not improve pathologic complete response rates in early, high-risk triple-negative breast cancer. In this trial, PD-L1 expression correlated with pathologic complete response [28].

#### 3.2.2. Avelumab

Avelumab is another PD-L1 inhibitor undergoing clinical development. In a phase Ib solid tumor basket trial (JAVELIN; NCT01772004), avelumab was used to treat an expansion cohort consisting of 168 metastatic breast cancer patients, with tumors that were unselected for PD-L1 status and breast cancer subtype [29]. The TNBC subtype represented 34.5% of patients. Approximately 50% of the TNBC patients had ≤1 prior regimen for metastatic disease. The ORR in the TNBC cohort was 8.6% (95% CI 2.9-19); 5 of 58 patients had a PR, and 13 had stable disease (22.4%) [29].

Avelumab is also under assessment in adjuvant setting in the A-BRAVE Trial (NCT02926196). It is a phase III randomized trial evaluating adjuvant treatment with avelumab in 335 patients with TNBC who completed definitive curative therapy [30].

#### 3.2.3. Durvalumab

In metastatic TNBC, durvalumab is being evaluated in combination with Vigil (autologous tumor cell immunotherapy; NCT02725489) [31] and in combination with paclitaxel (NCT02628132) [32], olaparib (NCT02484404) [33], tremelimumab (NCT02527434) [34], and epacadostat, an inhibitor of indoleamine 2,3-dioxygenase (NCT02318277) [35].

It was also tested in combination with other agents for early-stage TNBC.

The addition of durvalumab to anthracycline/taxane-based chemotherapy showed encouraging results as neoadjuvant therapy for early TNBC in the randomized phase II GeparNuevo study [36]. The primary endpoint was the pCR rate that was 53.4% in the durvalumab arm vs. 44.2% for chemotherapy alone (control arm), and the best response rates were observed when durvalumab was given for a window of 2 weeks before chemotherapy, priming the immune system first.

Another phase I/II neoadjuvant study (NCT02489448) of weekly nab-paclitaxel followed by dose-dense doxorubicin and cyclophosphamide with concurrent durvalumab in stages I-III TNBC showed that addition of durvalumab is safe and the pCR rates appear to be higher than what is expected with chemotherapy alone [37].

## 4. Future Perspectives

### 4.1. Combining Immune Checkpoint Inhibitors with PARP Inhibitors

So far, few clinical trials have explored the immunomodulatory potential of PARPi. However, recent data demonstrate the presence of a functional link between DNA damage response and anticancer immunity [38]. This link could be explained by the impact of genomic instability and tumor mutational burden on the production of tumor neoantigens and also by the immunogenic cell death induced by DNA damage.

Additionally, the activation of the “innate cytosolic immunity” signaling pathways is in response to DNA damage. This latter aspect was notably revealed by two recent studies which have demonstrated the capacity of certain DNA repair defects, present in the tumor, to stimulate an antitumor immune response by activation of the cGAS/STING (cyclic) pathway and GMP-AMP synthase/stimulator of interferon genes [39, 40].

Several trials have been conducted to explore the real effectiveness of combination therapy.

TOPACIO/KEYNOTE-162 is an open-label, single-arm phase 2 trial including 55 patients with advanced TNBC regardless of the BRCA mutation status or the programmed expression of the death ligand 1 (PD-L1) having received niraparib and pembrolizumab; the ORR was 21% and the disease control rate was 49%. In 15 patients with a BRCA tumor mutation, the ORR was 47% and the disease control rate was 80% [41].

Many other trials are assessing the combination of immune checkpoint inhibitors and PARP inhibitors.

A randomized phase II study is exploring the efficacy of olaparib or olaparib in combination with durvalumab in platinum-treated mTNBC NCT03167619 [42].

Another phase I/II is evaluating durvalumab in combination with olaparib and/or cediranib NCT02484404 [33].

Avelumab, another anti PD-L1, is under evaluation in association with talazoparib in phase II trial NCT03330405 [43].

Additionally, atezolizumab is also being explored with rucaparib in a phase I study NCT03101280 [44] and with olaparib in a phase II trial NCT02849496 [45].

### 4.2. Combining Immune Checkpoint Inhibitors with CDK4/6 Inhibitors

CDK4/6 inhibitors have been shown to be able to induce an antitumor immune response by different mechanisms, including increased presentation of the antigen by tumor cells, in addition to stimulation of the activation of effector T lymphocytes and reduction of proliferation of immunosuppressive Treg cells [46, 47].

The combination of CDK4/6 inhibitors with immune checkpoint inhibitors, targeting PD-1 and CTLA-4, has shown promising results in preclinical trials by inducing complete and lasting regressions (>1 year) in mouse models of established xenografts of human TNBC [48].

The rationale of the efficacy of the combination of CDK4/6 inhibitors and immunotherapy was based on different mouse models. Preliminary positive results from a phase Ib clinical trial studying abemaciclib with pembrolizumab in HER2-negative ER-positive MBCs showed an ORR of 14.3% in a 16-week interim analysis with a 75% of disease control rate [49]. Randomized clinical studies investigating the combination of CDK4/6 inhibitors and immunotherapy in the triple-negative subgroup are needed to shed light on the effectiveness of this strategy in this group of patients.

### 4.3. Adoptive T Cell Therapy

CAR T cells are genetically engineered T cells equipped with a tumor-specific chimeric antigen receptor. Antibody-derived chimeric antigen receptor (CAR) T cell therapy has demonstrated very promising results in hematologic malignancies with less success in solid tumors. Some research has shown that CAR T cells targeting tumor MUC1 glycoprotein may reduce the growth of TNBC [50]. The monoclonal antibody, TAB004, specifically recognizes the aberrantly glycosylated tumor form of MUC1 in all subtypes of breast cancer including 95% of TNBC while sparing recognition of normal tissue MUC1. MUC28z CAR T cells demonstrated significant target-specific cytotoxicity against a panel of human TNBC cells. Thus, MUC28z CAR T cells have high therapeutic potential against tMUC1-positive TNBC tumors with minimal damage to normal breast epithelial cells [50].

Additionally, encouraging results were seen with TGF-*β* that suppresses cytolytic capacity, cytokine production, and proliferation of CAR T cells against TNBC *in vitro*. These immunosuppressive effects can be neutralized by TGF-*β* receptor I kinase inhibition. Therefore, these findings encourage the evaluation of ROR1^+^ CAR T cells against TNBC in combination with a TGF-*β* inhibitor [51].

More recently, mesothelin, a cell surface glycoprotein normally present in mesothelial cells, was identified as a potential immunotherapy target [52]. This glycoprotein has already demonstrated encouraging results in mesothelioma and ovarian cancer. In a study including 99 primary breast cancers, it has been found that 67% of patients with TNBC expressed the mesothelin in at least 5% of tumor cells, with 19% of patients with TNBC expressing mesothelin in over 50% of tumor cells. In contrast, it was rarely expressed in luminal or HER2-positive breast cancer [52, 53]. These findings showed that mesothelin can be a promising target for adoptive T cell therapy of TNBC, but more advanced research is still needed to validate it.

Another molecule is under investigation in TNBC, which is an intercellular adhesion molecule-1 (ICAM-1). It has been found that this molecule is upregulated in TNBC and could and then may be a promising target in the future by evaluating the activity of ICAM-1-specific CAR T cells for patients with ICAM-1-positive TNBC [54].

### 4.4. Vaccines

The big advances of immunotherapy seen in TNBC led to push research about genetic vaccine against TNBC *in vitro*. It has been demonstrated that TNBC presents a high level of expression of Runx2 in TNBC comparing to other breast cancer subtypes.

A Runx2 lentivirus transfection system was successfully engineered, and Runx2 was transduced into dendritic cells while maintaining stable expression. The sustained and stable cytotoxic T cells induced in the transfected group had higher and more specific antitumor efficacy against TNBC, compared with the other cell lines. With these results, Runx2 is an attractive target in TNBC and development of Runx2-DC vaccine may induce specific and efficient activity in TNBC *in vitro* [55].

Cancer-testis antigens (CTAs) are a heterogeneous group of tumor-associated antigens (TAAs) present features that may be very attractive to be targeted by immunotherapeutic agents [56]. TNBC expresses several specific CTAs such as the MAGE group, SP17, and NY-ESO-1.

NY-ESO-1 expression was identified as an independent good prognostic factor in TNBC, and its expression was associated with higher humoral immune response and higher TILs [57]. Thus, the identification of tumors expressing NY-ESO-1 can allow the selection of patients with a higher potential of response to vaccination therapy.

SP17 is a protein expressed in breast cancer more importantly than in TNBC. The SP17-specific, HLA class I-restricted, cytotoxic T lymphocytes were associated with higher activity against breast cancer cells. Therefore, SP17 may be interesting in the development of breast cancer vaccines.

## 5. Conclusion

Immunotherapy has shown high efficacy in TNBC especially after the results from the IMpassion130 trial that has changed the standard of care in at least a subset of patients with metastatic PD-L1-positive TNBC. New strategies by the combination of immune checkpoint blockade with conventional therapies including chemotherapy, radiotherapy, and targeted therapies are also promising, and further research is needed to identify new biomarkers to select better responders to these treatments.

## Figures and Tables

**Table 1:** The main studies of immune checkpoint inhibitors in triple-negative breast cancer.

Disease setting	Trial	Phase	Drug	Line	Number of patients	PD-L1 status	Endpoints
Advanced PD-L1^+^ TNBC	KEYNOTE-012, NCT01848834	Ib	Pembrolizumab	≥1	27	PD-L1 expression in stroma or ≥1% of tumor cells	ORR: 18.5% 6-month PFS: 24.4% mPFS: 1.9 months mOS: 11.2 months 24-month OS: 22%
Cohort A: advanced, previously treated, any PD-L1 TNBC	KEYNOTE-086, NCT02447003	II	Pembrolizumab	Cohort A: >1	Cohort A: 170	Cohort A: —	Cohort A: ORR: 5.3% mPFS: 2 months mOS: 8.9 months
Cohort B: advanced, untreated PD-L1^+^ TNBC				Cohort B: 1	Cohort B: 84	Cohort B: CPS ≥ 1%	Cohort B: ORR: 21.4% mPFS: 2.1 months mOS: 18 months
Advanced TNBC unselected for PD-L1	ENHANCE-1, NCT02513472	Ib/II	Pembrolizumab+eribulin mesylate	≥1	82	—	ORR: 25.6% mPFS: 4.1 months mOS: NE
Metastatic, previously treated, PD-L1^+^ TNBC	KEYNOTE-119, NCT02555657	III	Pembrolizumab vs. chemotherapy	>1	622	CPS ≥ 1%	CPS ≥ 1%: mOS: 10.7 vs. 10.2 months (*p* = 0.073) CPS ≥ 10%: mOS: 12.7 vs. 11.6 months (*p* = 0.057)
Previously untreated, locally recurrent, inoperable TNBC	KEYNOTE-355, NCT02819518	III	Pembrolizumab+chemotherapy vs. placebo chemotherapy	1	847	—	CPS ≥ 10%: mPFS: 9.7 vs. 5.6 months (*p* = 0.0012) CPS ≥ 1%: mPFS: 7.6 vs. 5.6 months (*p* = 0.0014) ITT: mPFS: 7.5 vs. 5.6 months
Metastatic TNBC after induction treatment	TONIC, NCT02499367	II	Nivolumab	>3	*N* = 66 Cohort 1 (no induction): 12 Cohort 2 (radiotherapy): 12 Cohort 3 (cyclophosphamide): 12 Cohort 4 (cisplatin): 13 Cohort 5 (doxorubicin): 17	—	ORR: Overall: 20% Cohort 1: 17% Cohort 2: 8% Cohort 3: 8% Cohort 4: 23% Cohort 5: 35%
Advanced TNBC unselected for PD-L1	NCT01375842	I	Atezolizumab	≥1	115 1Line : 21 > 1Line : 94	—	ORR: 10% (1Line : 24% > 1Line : 6%, 12.7% in PD-L1^+^) mPFS: 1.4 months by RECIST, 1.9 by irRECIST mOS: 8.9 months (1Line : 17.6 months > 1Line : 7.3 months)
Advanced, untreated TNBC unselected for PD-L1	IMpassion130, NCT02425891	III	Atezolizumab+nab-paclitaxel vs. placebo+nab-paclitaxel	1	902 (1 : 1)	PD‐L1 ≥ 1%	ITT: mPFS: 7.2 vs. 5.5 months (*p* = 0.002) mOS: 21.3 vs. 17.6 months (*p* = 0.08) PD‐L1 ≥ 1%: mPFS: 7.5 vs. 5 months (*p* < 0.001) mOS: 25 vs. 15.5 months
Metastatic TNBC unselected for PD-L1	JAVELIN, NCT01772004	I	Avelumab	>1	168 (58 TNBC)	—	ORR: 5.2% (PD-L1^+^: 22.2% vs. PD-L1-: 2.6%) mPFS: 1.5 months mOS: 9.2 months
Neoadjuvant TNBC	I-SPAY 2, NCT01042379	II	Pembrolizumab+paclitaxel vs. paclitaxel	—	250 randomized: 69 experimental arms (29 TNBC) and 181 control arms	—	pCR: 60% vs. 20%
Neoadjuvant TNBC	KEYNOTE-522, NCT03036488	III	Pembrolizumab+chemotherapy vs. placebo+chemotherapy	—	1174 randomized: 784 experimental arms and 390 control arms	—	pCR: 64.8% vs. 51.2% (*p* = 0.00055)
Neoadjuvant TNBC	GeparNuevo, NCT02685059	II	Durvalumab+nab-paclitaxel vs. placebo+nab-paclitaxel	—	174	—	pCR: 53.4% vs. 44.2%
Neoadjuvant TNBC	NeoTRIP, NCT02620280	III	Atezolizumab+carboplatin+nab-paclitaxel vs. carboplatin+nab-paclitaxel	—	280	—	pCR: 43.5% vs. 40.8% (*p* = 0.066)

PD-L1^+^: expression in stroma or ≥1% of tumor cells by immunohistochemistry; CPS: combined positive score; ORR: objective response rate; OS: overall survival; PD-L1: programmed death-ligand 1; PFS: progression-free survival; RECIST: response evaluation criteria in solid tumors; irRECIST: immune-related response evaluation criteria in solid tumors; TNBC: triple-negative breast cancer; NE: not estimable; ITT: intention to treat; pCR: pathologic complete response.
